# A new method for identifying bivariate differential expression in high dimensional microarray data using quadratic discriminant analysis

**DOI:** 10.1186/1471-2105-12-S12-S6

**Published:** 2011-11-24

**Authors:** Jorge M Arevalillo, Hilario Navarro

**Affiliations:** 1 Department of Statistics and Operational Research, UNED, Paseo Senda del Rey 9, 28040 Madrid, Spain

## Abstract

**Background:**

One of the drawbacks we face up when analyzing gene to phenotype associations in genomic data is the ugly performance of the designed classifier due to the small sample-high dimensional data structures (*n* ≪ *p*) at hand. This is known as the peaking phenomenon, a common situation in the analysis of gene expression data. Highly predictive bivariate gene interactions whose marginals are useless for discrimination are also affected by such phenomenon, so they are commonly discarded by state of the art sequential search algorithms. Such patterns are known as weak/marginal strong bivariate interactions. This paper addresses the problem of uncovering them in high dimensional settings.

**Results:**

We propose a new approach which uses the quadratic discriminant analysis (QDA) as a search engine in order to detect such signals. The choice of QDA is justified by a simulation study for a benchmark of classifiers which reveals its appealing properties. The procedure rests on an exhaustive search which explores the feature space in a blockwise manner by dividing it in blocks and by assessing the accuracy of the QDA for the predictors within each pair of blocks; the block size is determined by the resistance of the QDA to peaking. This search highlights chunks of features which are expected to contain the type of subtle interactions we are concerned with; a closer look at this smaller subset of features by means of an exhaustive search guided by the QDA error rate for all the pairwise input combinations within this subset will enable their final detection. The proposed method is applied both to synthetic data and to a public domain microarray data. When applied to gene expression data, it leads to pairs of genes which are not univariate differentially expressed but exhibit subtle patterns of bivariate differential expression.

**Conclusions:**

We have proposed a novel approach for identifying weak marginal/strong bivariate interactions. Unlike standard approaches as the top scoring pair (TSP) and the CorScor, our procedure does not assume a specified shape of phenotype separation and may enrich the type of bivariate differential expression patterns that can be uncovered in high dimensional data.

## Background

The development of high-throughput technologies, such as gene or proteinn microarrays, has provided the scenario of the state of cells by monitoring the expression levels of hundreds or thousands of biological inputs (*p*) for a few number (*n*) of experimental units measured under different clinical conditions. A challenging problem within this domain is the identification of inputs or interactions of them highly correlated to the outcome. The low sample-high dimensional (*n* ≪ *p*) structure of the data we handle makes this challenge a difficult task, in particular when we are concerned with the detection of bivariate interactions. Some papers that tackle this problem using indexes of pairwise feature association are [1,2], which introduced the TSP score, and [3] which defined the CorScor index by the changes in the intra-class correlation coefficient and explores the feature space looking for gap/substitution and on/off association patterns. These approaches assume a specified shape for the interaction.

In this paper we address the problem by evaluating the performance of a classification rule trained on the data at hand; hopefully, this will enrich the typology of interactions that might be hidden in the data. One of the main drawbacks for facing up this problem is the well known peaking phenomenon; it consists of the deterioration of the performance of the designed classifier when the number of inputs increases and many noisy variables are involved in fitting the classifier, so the signal gets masked and the classifier confuses it with the noise. There is a great deal of literature discussing this phenomenon; some recent papers [4,5] study the problem within a general framework and [6] tackles it in the context of feature selection.

The peaking phenomenon is more acute for weak marginal/strong bivariate signals as pointed out in [7], that is, for highly predictive interactions having useless marginal distributions for classifying the outcome. This paper studies the peaking phenomenon for this type of bivariate interaction patterns. We propose a search procedure which utilizes the error rate of the quadratic discriminant analysis (QDA) classifier to carry out an exploration of the feature space in order to find such signals. The use of the QDA classifier will hopefully enlarge the type of patterns of bivariate differential expression uncovered by the aforementioned methods TSP and CorScor.

## Methods

### Motivation

This section gives a detailed description of how weak marginal/strong bivariate interactions are lost by different classification rules as the dimension of the feature space increases and many noise predictors are involved in training the classifier. For the sake of simplicity we confine ourselves to the binary classification problem, where *n*_0_ and *n*_1_ observations are drawn from each one of the categories of the outcome variable.

#### Weak marginal/strong bivariate interactions

Four examples of weak marginal/strong bivariate interaction patterns are given by the following synthetic scenarios.

### Scenario 1

The observations are drawn from bivariate random variable *Z* = (*Z*_1_, *Z*_2_) in accordance with the following scheme: the conditional distribution of *Z*|*Y* = 0 —black labels— is bivariate normal with mean vector *µ*_0_ = (0, 0); meanwhile, the other class conditional distribution for *Z*|*Y* = 1 —red labels— is bivariate normal with mean vector *µ*_1_ = (–1,1). We assume both distributions have the same covariance matrix given by

### Scenario 2 (XOR)

The observations for the XOR pair are drawn from a bivariate random variable *X* = (*X*_1_, *X*_2_) in accordance with the following scheme: the conditional distribution of the random variable *X*|*Y* = 0 —black labels— is uniform over the quadrants . On the other hand, *X*|*Y* = 1 —red labels— has a uniform distribution over .

### Scenario 3 (circular pattern)

Cases are simultated from a bivariate normal distribution *R* = (*R*_1_, *R*_2_) with vector means *µ* = (0, 0) and covariance matrix the indentity *I*. The labels are assigned in accordance to the following rules: if  then *Y* = 0 —black labels— and if  then *Y* = 1 —red labels.

### Scenario 4 (V-shaped pattern)

Observations in this situation are drawn from uniform distributions confined to the domain . The interaction between the pair *V* = (*V*_1_, *V*_2_) and the outcome variable *Y* is given by the following rules: *V*|*Y* = 0 —black labels— has a uniform distribution over , with . On the other hand, *V*|*Y* = 1 —red labels— has a uniform distribution over , with .

**Figure 1:**
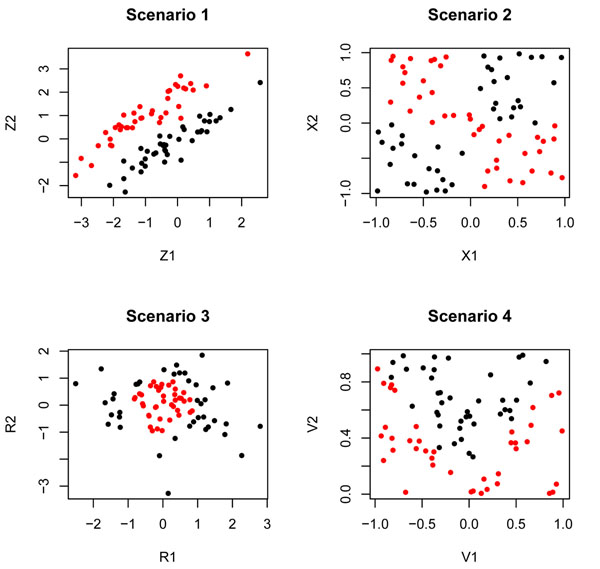
**Weak marginal/strong bivariate signals.** The figure displays the scatter plots for 80 observations —with sample sizes *n*_0_ = *n*_1_ = 40— generated in accordance to the linear, XOR, circular and V-shaped patterns defined in scenarios 1, 2, 3, and 4. The components of the bivariate interaction are weak for class prediction when considered individually; however, they exhibit a high predictive strength when taken jointly.

Figure 1 shows the scatter plots obtained by simulating observations in accordance to the four schemes for sample sizes *n*_0_ = *n*_1_ = 40. Note that if the points are projected on each one of the axes, both categories of the outcome do overlap; however, if both variables of the pair are considered together the classes are neatly separated. Therefore, the discrimination comes from the bivariate interaction between them. This is the reason why we call this type of interactions weak marginal/strong bivariate interactions.

#### The peaking phenomenon

**Figure 2:**
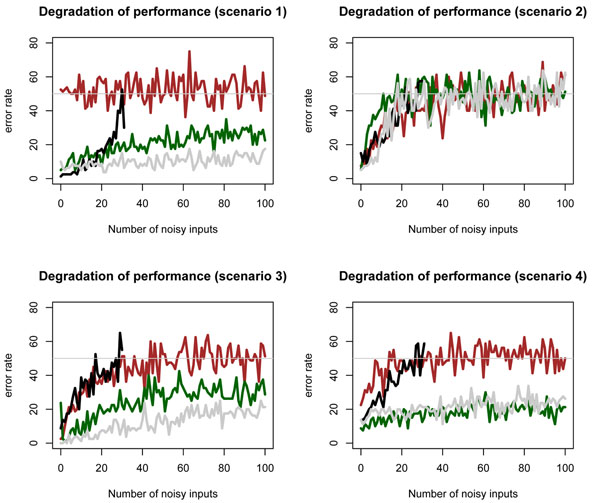
**Degradation of the performance for different classifiers.** Performance of SVM with polynomial kernel (brown curve), Random Forests (green curve), QDA (black curve) and Adaboost (gray curve), estimated by 10-fold cross validation (cv), as an increasingly number of noisy features is added to the patterns defined in scenarios 1, 2, 3 and 4 (left to right and top to bottom).

We consider the previous synthetic scenarios and generate samples of sizes *n*_0_ = *n*_1_ = 40. For each scenario, we add *j* independent noisy features, *j* = 1, 2,…, 100, with standard normal distribution and estimate the error rate for the following four classification rules: Adaboost [8], Random Forests (RF) [9], a support vector machine (SVM) with polynomial kernel [10] and the QDA classifier [11] . The error rate is estimated by 10-fold cross validation. The results are shown in the plots of Figure 2.

Note that the error rate of the polynomial kernel in scenario 1 is high, around 0.5, when only the variables of the pair (*Z*_1_, *Z*_2_) are used as predictors. This ugly performance is related to the type of polynomial kernel we used, a second order polynomial kernel, which is well suited for tracing nonlinear quadratic patterns but poor for identifying linear decision boundaries as in scenario 1; see the improvement of SVM classifier in scenarios 2, 3 and 4 where it makes a better job with the non linear interaction patterns (*X*_1_, *X*_2_), (*R*_1_, *R*_2_) and (*V*_1_, *V*_2_) tracing the separation between the classes.

In addition, we can observe that the error rate deteriorates as the number of features increases; this shows the peaking phenomenon for weak marginal/strong bivariate interactions. The QDA resistance to peaking compares to RF and Adaboost, with the exception of scenario 3 when *p* < 20; see that the error rates are nearly similar, specially for a number of inputs under 10 or 15. In addition, the QDA has the appealing property of requiring a low computational cost for training the classifier; this fact is crucial in the design of the search strategy since our procedure will explore the feature space in an almost exhaustive way by fitting thousands of times the QDA classifier.

#### Comparative study for a benchmark of classification rules

The CMA package [12] from Bioconductor project repositories in [13] provides an interface for the analysis of genomic data. One of the utilities of CMA is the possibility to carry out a comparative study of the performance of classifiers for a benchmark of classification rules.

In this section we revisit the effect of the peaking phenomenon for a selection of classifiers from the CMA package: k-nearest neighbors (knn) and neural networks (nnet) [14], diagonal (DLDA), linear (LDA) and quadratic (QDA) discriminant analysis as in [11], partial least squares with lda, logistic regresion and RF variants (*pls_lda*, *pls_lr*, *pls_rf*) as in [15], PAM classifier (scDA) as introduced in [16], random forests [9], the componentwise boosting (compBoost) introduced in [17], the ElasticNet [18] and two versions of the SVM (svm, svm2) with second order polynomial and radial kernels respectively. The error rate was estimated by 10-fold cross validation.

The function compare gives a picture of how these classifiers compare one with each other. For each one, it displays the boxplot of the error rate over the 10 validation sets.

**Figure 3:**
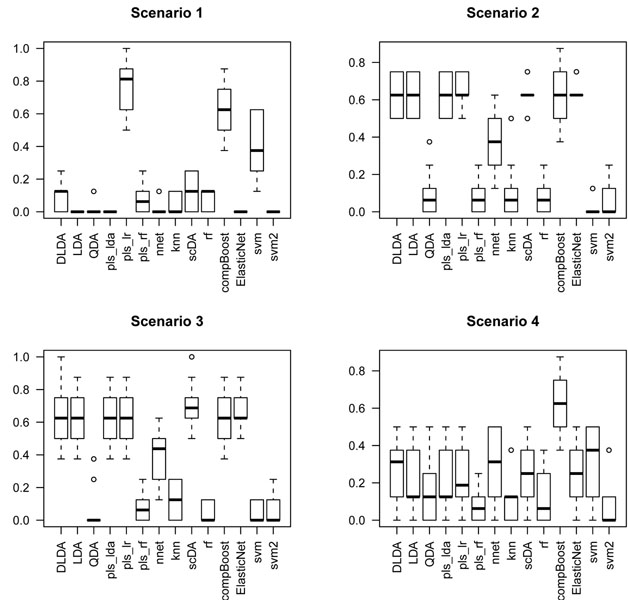
**Boxplots of error rate over the 10 validation sets: p = 2.** The figure displays boxplots of the performance of a benchmark of classification rules by measuring the error rate over the 10 cross validation sets when only the bivariate patterns of scenarios 1, 2, 3 and 4 form the feature space. The plots were obtained by the compare function of CMA bioconductor package.

**Figure 4:**
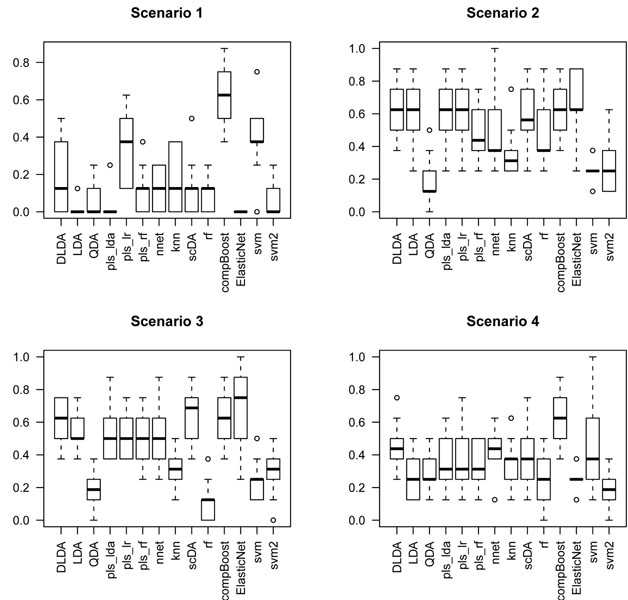
**Boxplots of error rate over the 10 validation sets: p = 10.** The figure displays boxplots of the performance of a benchmark of classification rules by measuring the error rate over the 10 cross validation sets when eight noisy variables along with the bivariate patterns of scenarios 1, 2, 3 and 4 form the feature space. The plots were obtained by the compare function of CMA bioconductor package.

**Figure 5:**
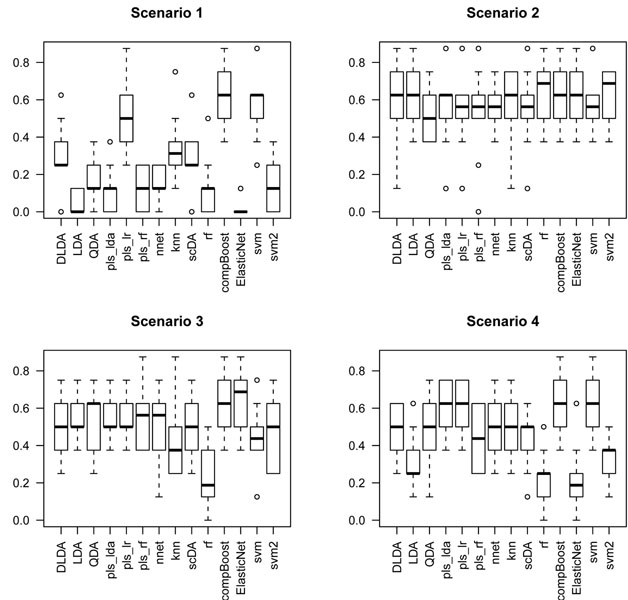
**Boxplots of error rate over the 10 validation sets: p = 20.** The figure displays boxplots of the performance of a benchmark of classification rules by measuring the error rate over the 10 cross validation sets when eighteen noisy variables along with the bivariate patterns of scenarios 1, 2, 3 and 4 form the feature space. The plots were obtained by the compare function of CMA bioconductor package.

The data sets were generated by drawing *n*_0_ = *n*_1_ = 40 samples from scenarios 1, 2, 3 and 4. The boxplots in Figures 3, 4 and 5 give a glance of the performance of the classifiers. The experiment was carried out for *p* = 2 (the signal alone), *p* = 10 (signal and 8 noisy inputs) and *p* = 20 (signal and 18 noisy inputs) features. We can see that QDA outperforms the remaining classification rules in almost all the scenarios. It is of special interest the XOR interaction pattern as pointed out in [3]; in this case, all these simulations have shown that the performance of all the classifiers deteriorates when the number of features reaches *p* = 20. It is worth noting that for *p* under 10 (see Figure 4) the most resistant classifier to peaking for the XOR signal is QDA; meanwhile, for *p* = 20 all the classifiers are highly affected by peaking in the XOR scenario (see scenario 2 in Figure 5). So we conclude that QDA is a good candidate for designing a search strategy that uncovers this type of interaction patterns.

#### A closer look at QDA and its resistance to peaking

Now we explore with more detail the resistance of QDA classification rule to peaking by means of a simulation study.

Recall that binary QDA is concerned with the discrimination between two *p*-dimensional multivariate normal class conditional populations *N*(*µ*_1_, Σ_1_) and *N*(*µ*_2_, Σ_2_), where *µ*_1_ and *µ*_2_ are the mean vectors and Σ_1_ and Σ_2_ are the covariance matrices. The decision boundary corresponding to the QDA classification rule is given by

with  and *π*_1_, *π*_2_ the a priori class probabilities. When we are dealt with balanced classes *π*_1_ = *π*_2_ = 0.5.

When the sample estimates , , ,  of the covariances and the means are plugged in the expression above, we obtain the QDA designed classifier.

The previous decision boundary defines an hyperquadric whose shape depends on the elements involved in the difference of inverses , more specifically on the product of its eigenvalues. This yields to elliptical, hyperbolic, parabolic or linear boundaries; see [19] for details. Thus, the variety of patterns recognized by QDA is rich enough to consider it a good classification rule for pattern discovery. We now carry out a simulation experiment in order to study its resistance to peaking for weak marginal/strong bivariate interactions.

We have drawn 80 observations (*n*_0_ = *n*_1_ = 40) according to patterns in scenarios 1, 2, 3 and 4, along with 80 cases from *p –* 2 independent standard normal variables, which are uninformative features for class prediction. On the order hand, we have generated *n*_0_ + *n*_1_ = 80 samples from *p* independent standard normal variables and obtain a data set with only noisy features. The error rate of the QDA classifier was estimated by 10-fold cross validation for each data set. We repeated the experiment *B* = 100 times in order to get both populations of error rates: with the signal and with only noisy features. We have considered feature spaces with *p* = 2, 5, 10, 15, 20, 30 predictors.

**Figure 6:**
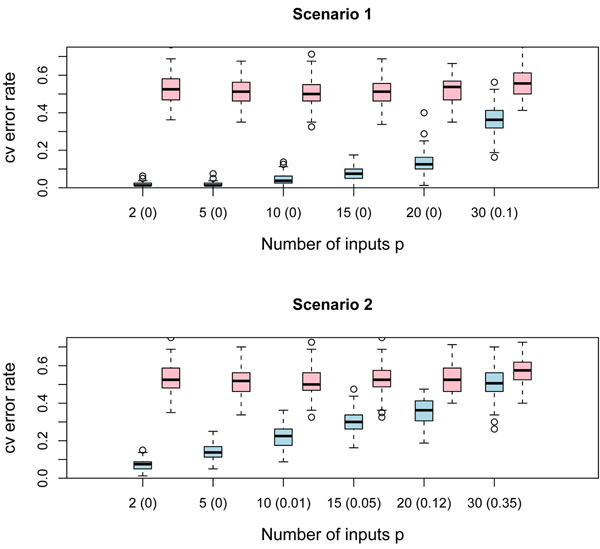
**Boxplots of QDA performance. Scenarios 1 and 2.** The figure displays boxplots of the QDA 10-fold cross validation error rate for 100 simulations of the following experiment: 80 observations, with class sizes *n*_0_ = *n*_1_ = 40, were generated for different dimensions *p* = 2, 5, 10, 15, 20, 30 of the feature space for chunks of noisy variables and once again for chunks containing the weak marginal/strong bivariate signal. The QDA error rate is computed in both cases. Boxplots in blue correspond to the cv QDA error rate for chunks with the weak marginal/strong bivariate signal; on the other hand, boxplots in pink correspond to the cv QDA error rate for blocks containing only noisy features. The amount of overlap between both populations of boxplots is shown in parenthesis. The simulation was carried out for the weak marginal/strong bivariate signals of scenarios 1 and 2.

**Figure 7:**
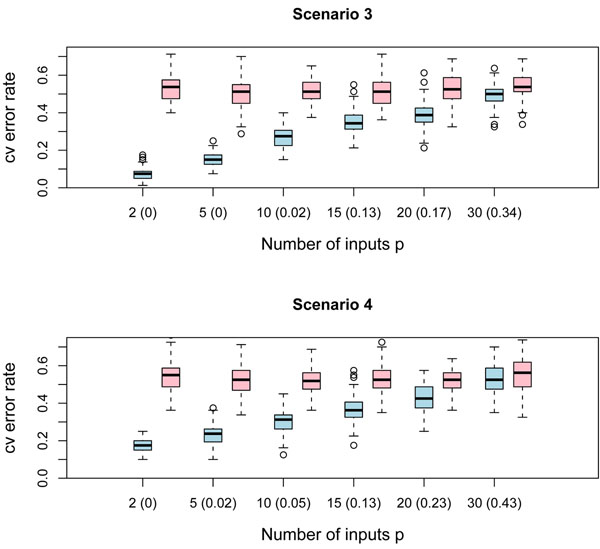
**Boxplots of QDA performance. Scenarios 3 and 4.** The figure displays boxplots of the QDA 10-fold cross validation error rate for 100 simulations of the following experiment: 80 observations, with class sizes *n*_0_ = *n*_1_ = 40, were generated for different dimensions *p* = 2, 5, 10, 15,20, 30 of the feature space for chunks of noisy variables and once again for chunks containing the weak marginal/strong bivariate signal. The QDA error rate is computed in both cases. Boxplots in blue correspond to the cv QDA error rate for chunks with the weak marginal/strong bivariate signal; on the other hand, boxplots in pink correspond to the cv QDA error rate for blocks containing only noisy features. The amount of overlap between both populations of boxplots is shown in parenthesis. The simulation was carried out for the weak marginal/strong bivariate signals of scenarios 3 and 4.

The boxplots of Figures 6 and 7 show the results of the simulations. The amount of overlap between both populations is shown for each *p* in parenthesis. This overlap was measured by one of the alternatives widely used in the statistical practice: the well known measure Φ(–∆/2), with Φ the distribution function of the standard normal variable and ∆ the Mahalanobis distance. This measure has an appealing theoretical flavor since it provides the overall error rate of the linear discriminant classifier when the multivariate normality within the classes and the equality of class covariance matrices assumptions are met; see [11]. Note that the amount of overlap between both populations is always less than 5% when the number of predictors is smaller than 10, which means that QDA classification rule is able to distinguish between chunks of inputs containing a weak marginal/strong bivariate signal and chunks with only noisy features, provided that the size of the chunk is not greater than 10. As *p* increases, the amount of overlap becomes larger; therefore the QDA would be unable to catch the signal and might confuse it with the noise.

### The QDA interaction detector procedure

The results of the simulations have shown that QDA resistance threshold to peaking can be set at *p* = 10 (or at most *p* = 15), when we are concerned with the detection of weak marginal/strong bivariate interactions in high dimensional data sets. This finding is crucial and puts the basis of the search strategy we will design to uncover this type of interaction patterns. The rationale behind this strategy is as follows. The naive solution would explore the feature space in an exhaustive way by fitting a QDA classifier to each pair of variables; a high accurate classification would be highlighting the presence of a signal. Obviously, this alternative is time consuming prohibitive since it would require a total of *p*(*p* – 1)/2 QDA fits; for example, if *p* = 2000 then 1999000 fits are needed.

Our search strategy proceeds in a nearly exhaustive way by dividing the feature space in small blocks of features of a specified size *bsize* and by fitting the QDA for all pairs of blocks. As we know that QDA is resistant to peaking while the number of features ranges between p* = 10 and p* = 15, we propose to take *bsize* such that 2 ∗ *bsize* ≤ p*; in this way we protect ourselves against the danger of peaking when the QDA classifier is fit with all the features belonging to the union of both blocks. Once the QDA classifiers are obtained for all the possible matchings of blocks, we know that for a matching containing a bivariate interaction pattern, the classifier will give a very low error rate; meanwhile, for a block matching with only noisy features we will obtain a high error rate. Thus, we can construct a ranking of block matchings, where the top ranked matchings will contain the informative bivariate interaction patterns, and the matchings at the bottom of the ranking carrying on only noisy features. Now, at a second stage we can restrict the search to the subset of features belonging to the top ranked matchings of blocks. For example if we confine the search to the 2 ∗ *bsize* features of the first block matching, we would need to explore *bsize* × *bsize* interactions in order to find out which one of them is responsible for the observation of such a low error rate in the QDA; usually this search is very low time consuming since *bsize* is smaller than 7.

Searching in the feature space in a blockwise manner has an enormous advantage with respect to the exhaustive search; for example, if *p* = 2000 and we take *bsize* = 5, we would obtain 400 blocks; so the search would need only 79800 QDA fits, much less than the 1999000 fits of the naive solution.

This procedure has been implemented using the R platform for statistical computing downloaded from [20]. The following steps summarizes the main stages.

It is recommended to carry out a first screening step in order to filter the strong marginal features highly correlated with the outcome before applying the search procedure. Recall that our search strategy was designed to uncover the weak marginal/strong bivariate interactions which are usually rejected by traditional sequential search procedures or pre-screening filtering tools (see [7] for a detailed explanation of this fact).

## Results and discussion

### A simulation example for synthetic data

Let *n*_0_ = *n*_1_ = 40 be the class sizes. The cases were drawn from *p*-dimensional random vectors, (*Z*_1_, *Z*_2_, *E*), (*X*_1_, *X*_2_, *E*), (*R*_1_, *R*_2_, *E*) and (*V*_1_, *V*_2_, *E*) corresponding to scenarios 1, 2, 3 and 4, with *E* = (*E*_1_, …, *E_p_*_–2_) a vector of independent noisy standard normal variables added to the signal. For *p* = 200 the signal represents 1% of a 200-dimensional feature space.

**Figure 8:**
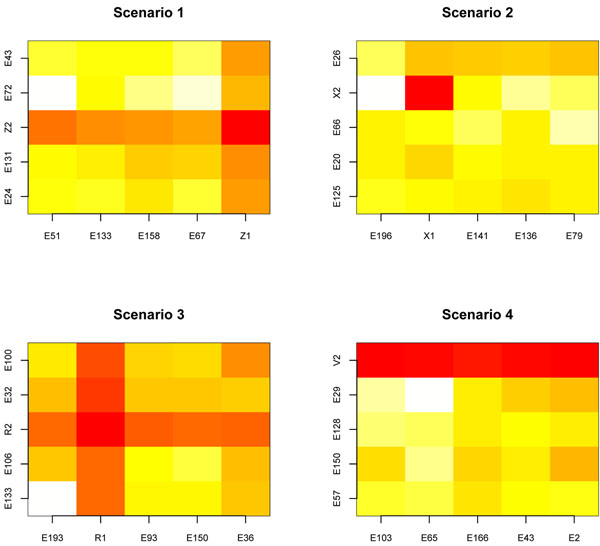
**Heat matrix for QDA cross validation error rate. Results for synthetic data.** Results for synthetic data The figure displays the heat matrix of the 10-fold cross validation error rate of the QDA classification rule, obtained for all pairwise combinations of features belonging to the first position of the ranking of block matches. Four synthetic data containing the weak marginal/strong bivariate signals of scenarios 1, 2, 3 and 4, along with 198 noisy features, were generated. The signal represents 1% of the dimension of the feature space. The red color is a hot spot corresponding to a low error rate and a fancy interaction; meanwhile light yellow spots represent large error rates corresponding to pairs of noisy features.

We have applied the QDA interaction detector procedure with *bsize* = 5 to the previous synthetic scenarios and have obtained the ranking of block matchings. As we have discussed, this ranking is a useful tool that allows to restrict the search for the hidden interaction patterns by exploring its top ranked positions. Figure 8 displays the heat matrix of QDA errors for all the bivariate interactions obtained from the first position of the ranking of block matchings. Light yellow and orange shades represent a high error rate while the red color represents a low error rate.

Note that the procedure has found interaction patterns (*Z*_1_, *Z*_2_), (*X*_1_, *X*_2_) and (*R*_1_, *R*_2_). The red hot squares of scenarios 1, 2, 3 and 4 in Figure 8 highlight the weak marginal/strong bivariate interaction hidden in the matching. These matrices may also provide a useful tool for identifying different types of weak marginal/strong bivariate signals: for example the first row of red squares in scenario 4 is highlighting the not so weak behavior of component *V*_2_ in the V-shaped pattern. On the other hand, the isolated red hot spot in scenario 2, surrounded by light yellow and orange spots, might be explaining the weak predictive power of the components in the XOR interaction, where both variables behave as noisy features.

### An application to real data: the colon cancer dataset

The colon cancer data set is a publicly available experiment which can be obtained from the package colonCA in [20]. Gene expression levels for 2000 genes across 40 tumor and 22 normal tissue samples were collected with Affymetrix oligonucleotide arrays [21]. The data were preprocessed by a log transformation and standardization across genes.

Random Forests (RF) outlier detector utility identified cases 18, 20, 52, 55 and 58 as outliers. These were previously identified in [22] as aberrant observations and will be removed from the analysis.

### Data analysis and findings

**Figure 9:**
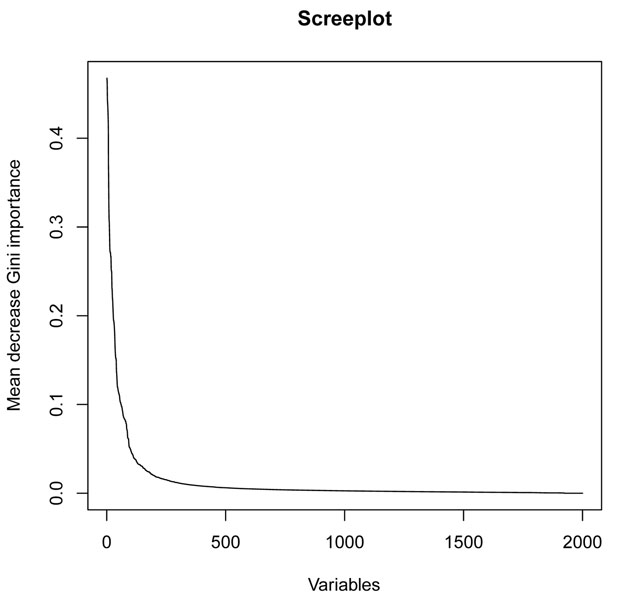
**Screeplot for Random Forests (RF) index of variable importance.** The screeplot of variable importance score for the genes from the colon cancer data as provided by RF ranking table of variable importance. The elbow is located at position 100 which suggests a first selection of one hundred biomarkers which are put aside at this screening stage.

The table of variable importance of RF identifies the most influential genes for class prediction. We took as a measure of importance the mean decrease Gini score; we utilized the values *ntree* = 5000 for the number of trees in the forest and the default for *mtry*, the number of eligible splitters. Figure 9 shows the screeplot for the variable importance scores. After a deep decay, we find a long plateau which begins at an elbow located at position 100 of the ranking.

We pick up the first one hundred genes of RF table of variable importance. The list has a great agreement with other previous selections in the literature, in particular with that one in [23]. Table 1 shows the identifiers of the genes of the list (four biomarkers identified as control are not reported). We put them aside and retain the remaining ones for the application of QDA interaction detector procedure.

**Table 1:** Subset of genes retained from a previous Random Forests screening step

M76378	M63391	M76378	M36634	R87126	J02854	Z50753
M76378	H43887	T92451	J05032	R36977	X12369	X63629
T71025	H40095	Z49269	R44301	M22382	X14958	U25138
R78934	H06524	T86473	H77597	H64489	M64110	X12671
Z49269	X86693	L05144	U19969	M26697	T40454	H20709
X54942	T51534	X16356	X70326	R42501	X87159	D25217
Z24727	R08183	L07648	H08393	U31525	M36981	M26383
X74295	T51571	R48303	T95018	T67077	M80815	U22055
T86749	R46753	X07290	T51539	T60155	U17899	U32519
D31716	H20426	D16294	U09564	R28373	R64115	X12466
R44418	X53743	U14631	X53461	R37276	D31885	X56597
T96873	X15882	T94350	X12496	D59253	D29808	R75843
L41559	T40645	M69135	U26312	T51858	R60883	R84411
Z25521	M26683	D42047	D15049	D14662		

#### Application of the QDA interaction detector procedure

After putting aside the biomarkers identified in the screening step and eliminating a few duplicated columns, we end up with a data set containing 1891 features along with the binary outcome.

**Figure 10:**
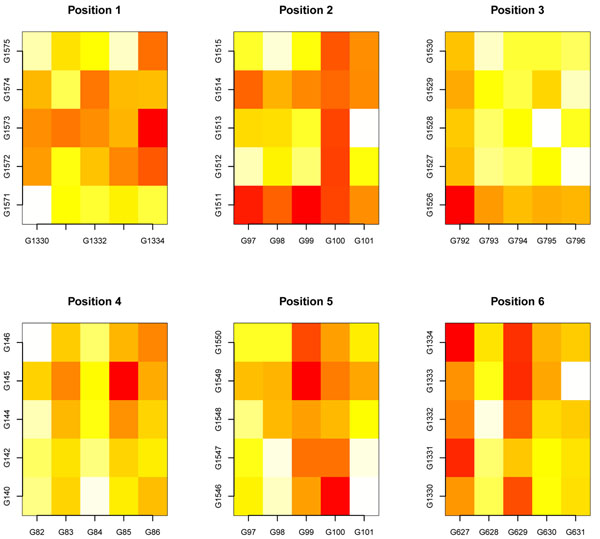
**Heat matrix for QDA cross validation error rate. Results for the colon cancer data set.** The figure displays the heat matrices of the 10-fold cross validation error rate of the QDA classification rule, obtained for all pairwise combinations of features belonging to the first six positions of the ranking of block matchings when the method is applied to the colon cancer data set. The red color is a hot spot corresponding to a low error rate and reveals a fancy gene to gene interaction; meanwhile light yellow spots represent large error rates corresponding to uninformative gene interactions.

Before applying the procedure, we set *bsize* = 5. Figure 10 displays the heat matrix plots of the error rate given by QDA classification rule for all the bivariate associations obtained by pairwise matching of the variables belonging to the six top positions of the ranking of block matchings.

**Figure 11:**
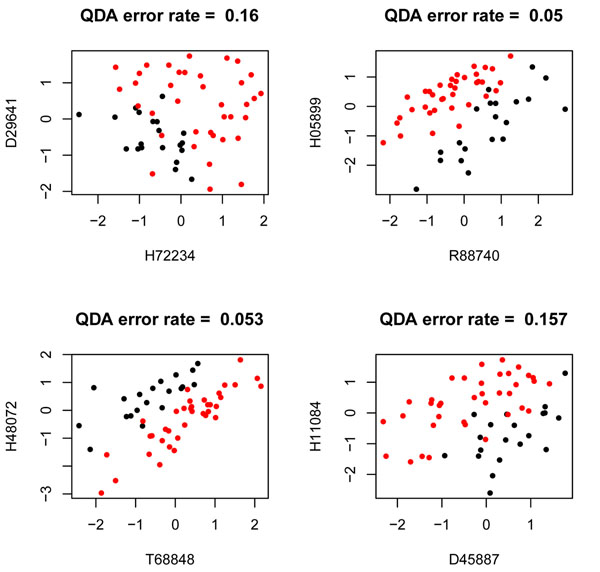
**Scatter plots for the selection given by the QDA interaction detector procedure.** The figure shows the scatter plots of the best gene to gene interactions: (*H*72234; *D*29641), (*R*88740; *H*05899), (*T*68848; *H*48072) and (*D*45887; *H*11084) provided by the QDA interaction detector procedure.

The heat matrix plots reveal interesting interactions among features which were considered useless by RF ranking of variable importance at the initial screening step. Four bivariate interactions are standing out; they correspond to the interaction of the genes at columns: (*G*1334, *G*1573), (*G*792, *G*1526), (*G*85, *G*145) and (*G*99, *G*1549) which come from the first, third, fourth and fifth positions of the ranking of block matchings. These associations correspond to the following gene to gene interactions: (*H*72234, *D*29641), (*R*88740, *H*05899), (*T*68848, *H*48072) and (*D*45887, *H*11084). The scatter plots in Figure 11 contain the type of bivariate interaction pattern displayed by each one of them.

Note that none of the genes in the scatterplots exhibit univariate differential expression since both classes of the outcome do overlap when projected on the axes. However, if both genes are considered together, they discriminate the binary outcome; such discrimination stems from the bivariate association between them. They are four cases of weak marginal/strong bivariate gene interaction patterns uncovered by our procedure.

#### Comparative analysis with TSP and CorScor methods

**Figure 12:**
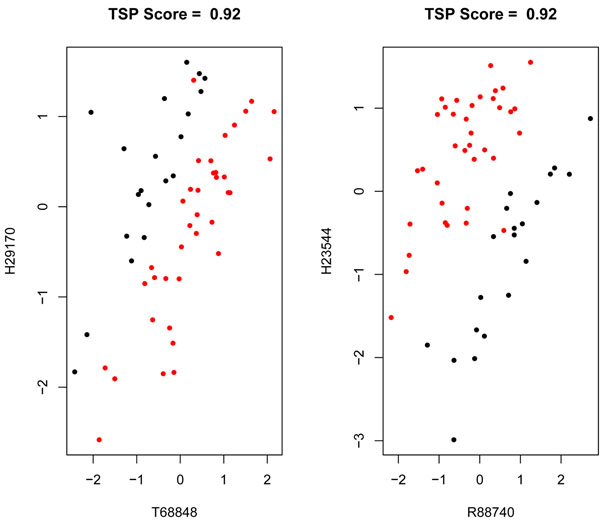
**Scatter plots for the best two top scoring pairs.** The figure shows the scatter plots of the best gene to gene interactions, (*T*68848; *H*29170) and (*R*88740; *H*23544), as provided by the TSP index.

**Figure 13:**
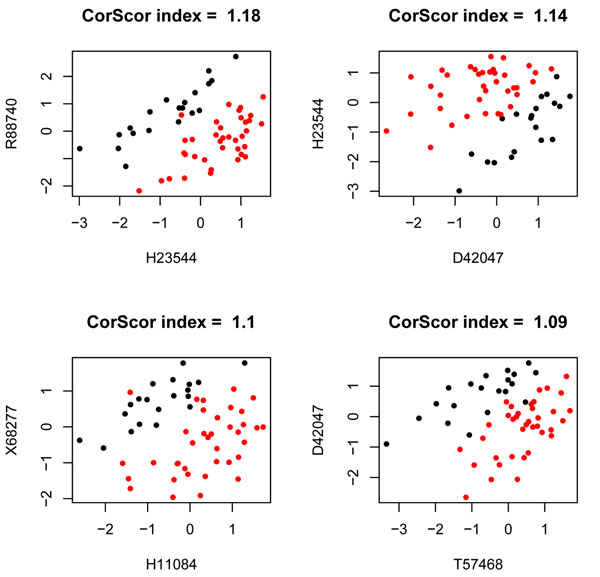
**Scatter plots for the best CorScor pairs.** The figure shows the scatter plots of the best four gene interactions: (*H*23544; *R*88740), (*D*42047; *H*23544), (*H*11084; *X*68277) and (*T*57468; *D*42047) given by the gap/substitution scenario of the CorScor index.

Table 2 shows the bivariate selection as provided by our procedure and the TSP and CorScor methods; it also provides the scores of the selected genes given by TSP and CorScor indexes. These results bring out a distinguishing issue, perhaps the most meaningful one, between the proposed method and some of the existing alternatives in the literature as TSP or CorScor indexes. The latter are identifying the interaction of genes (*R*88740, *H*23544) as a high scored one. The scatter plots in Figures 12 and 13 corroborates that both genes of the pair don’t exhibit univariate differential expression; however, a remarkable pattern of class separation stems from their association, which is described by a nearly linear decision boundary. This situation, although being very important for the aim of these two procedures, does not play the key role in our proposal which tries to detect, by means of a wider in purpose procedure, interactions which are not necessarily described in terms of such linear patterns of class separation. The connection between our method and the aforementioned TSP and CorScor is seen with the interactions (*R*88740, *H*05899), (*T*68848, *H*48072) and (*D*45887, *H*11084) where an almost linear pattern of class separation can be traced, although not so neatly as with the pair (*R*88740, *H*23544). Unlike them, the pair (*H*72234, *D*29641), with moderate to low values of the TSP and CorScor, exhibit a non linear pattern of class separation and is an example of the distinguishing feature between these methods and our approach.

**Table 2:** Selection of genes given by the methods: QDA interaction detector procedure, TSP and CorScor

QDA interaction detector procedure	TSP selection	CorScor selection
(H72234, D29641) *TSP* = 0.11 *CorScor* = 0.73	(T68848, H29170) *TSP* = 0.92 *CorScor* = 0.77	(H23544, R88740) *TSP* = 0.92 *CorScor* = 1.18

(R88740, H05899) *TSP* = 0.84 *CorScor* = 1.05	(R88740, H23544) *TSP* = 0.92 *CorScor* = 1.18	(D42047, H23544) *TSP* = 0.81 *CorScor* = 1.14

(T68848, H48072) *TSP* = 0.89 *CorScor* = 0.81		(H11084, X68277) *TSP* = 0.81 *CorScor* = 1.10

(D45887, H11084) *TSP* = 0.76 *CorScor* = 0.80		(T57468, D42047) *TSP* = 0.84 *CorScor* = 1.09

**Gene IDs**	**Gene descriptions**	

H72234	DNA-(APURINIC OR APYRIMIDINIC SITE) LYASE (HUMAN)	
D29641	Human mRNA (KIAA0052) for ORF, partial cds	
R88740	ATP SYNTHASE COUPLING FACTOR 6, MITOCHONDRIAL PRECURSOR (HUMAN)	
H05899	HETEROGENEOUS NUCLEAR RIBONUCLEOPROTEINS C1/C2 (HUMAN)	
T68848	PEPTIDYL-PROLYL CIS-TRANS ISOMERASE A (HUMAN)	
H48072	CYTOCHROME C OXIDASE POLYPEPTIDE VIA-LIVER (HUMAN)	
D45887	Human mRNA for calmodulin, complete cds	
H11084	VASCULAR ENDOTHELIAL GROWTH FACTOR (Cavia porcellus)	
H29170	ATP SYNTHASE B CHAIN, MITOCHONDRIAL PRECURSOR (HUMAN)	
H23544	GTP-BINDING NUCLEAR PROTEIN RAN (Homo sapiens)	
D42047	Human mRNA (KIAA0089) for ORF (mouse glycerophosphate dehydrogenase-related), partial cds	
X68277	H.sapiens CL 100 mRNA for protein tyrosine phosphatase	
T57468	FIBRILLARIN (HUMAN)	

## Conclusion

This paper has explored the peaking phenomenon in the context of detecting marginal/strong bivariate interactions in high dimensional settings. The appealing properties of the QDA classifier and its resistance to peaking has justified its use as a search engine of a procedure that explores the feature space in order to look for this type of signals in high dimensional data.

The method was applied both to artificial data and to a real microarray gene expression experiment, the colon cancer data set. The application to real data has led to promising results providing gene interactions that exhibit bivariate differential expression but are not differentially expressed when considered marginally. The results show the usefulness of QDA interaction detector procedure, which is expected to become an efficient tool for biologists and bioinformaticians for the discovery of new gene to gene interactions.

The proposed method has been developed for binary classification; the analysis for multi-class problems is a natural extension for conducting future research efforts. Some research regarding the computational cost involved in the QDA interaction detector search strategy is also an issue for further improvements.

## Competing interests

The authors declare that they have no competing interests.

## Authors’ contributions

The authors JMA and HN have equally contributed to the work. Both authors have read, reviewed and approved the final version of the manuscript.
